# JMIR Neurotechnology: Connecting Clinical Neuroscience and (Information) Technology

**DOI:** 10.2196/41122

**Published:** 2022-08-11

**Authors:** Pieter Kubben

**Affiliations:** 1 Faculty of Health, Medicine and Life Sciences School for Mental Health and Neuroscience Maastricht University Medical Center Maastricht Netherlands

**Keywords:** neurotechnology, neurological disorders, treatment tools, chronic neurological disease, information technology

I am happy to announce the launch of a new member of JMIR Publications’ open access family of journals, *JMIR Neurotechnology* (JNT). We are now accepting articles for submission and are waiving open access fees until we are indexed in PubMed.

## Why JNT Now?

Neurological diseases are an increasing concern for modern societies around the planet as rates of related disabilities and mortality have risen steadily over the past 2 decades [1]. Neurological disorders lead to significant reductions in the quality of life of those they afflict [2]. They are also clearly associated with steadily increasing costs of diagnosis and patient care, with estimates varying between US $650 and $800 billion per year [3]. Recent and numerous studies worldwide have confirmed that neurological diseases are becoming more widespread and chronic, and more expensive to treat and manage [1,2,4,5]. Such increases heighten the urgent need for more quality research coupled with the development of affordable tools to help diagnose and treat these complex and multivariate disorders.

Many areas of research and development come into play in the process of diagnosing and treating the expanding array of neurological disorders. Advances in many domains are providing valuable contributions, ranging from new findings from basic research aimed at unraveling fundamental, underlying neurological mechanisms to the evolving recognition of the potential of applied behavioral approaches (eg, mindfulness and meditation training) to improve neurological functions and health-related quality of life. Many of these advances are anchored in novel neurotechnology that provides both scientists and patients with new tools for research and treatment. In this context, we pragmatically define neurotechnology as the use of information technology to diagnose or treat chronic neurological disease.

Building on the JMIR foundation, JNT intends to support the development of novel diagnostic and treatment tools and paradigms for neurological diseases, leveraging recent insights from clinical neuroscience and information technology. Our hope is to foster the explicit linking of two domains into a merged domain and a single, coherent neurotechnology community, pragmatically defined as those exploring the use of technologies to diagnose and treat chronic neurological diseases. This community has formed as improvements in both hardware and software have paved the way for new paradigms in diagnostics and treatment through the merger of the clinical and information engineering worlds.

## The Aims and Scope of JNT

JNT aims to be a platform where applied human research can connect patients, caregivers, and information engineers active in any neurological domain. The journal editors welcome and will consider work in all relevant *clinical* domains including, but are not limited to, neurology, neurosurgery, neurorehabilitation, neuroradiology, and beyond. We also encourage the submission of work exploring the needs of professional and informal caregivers, all of whom need practical tools to inform and support patients in managing their neurological challenges. We hope that the journal will serve as a gathering place for those involved in patient care, whether directly or indirectly, since we believe that understanding and managing chronic diseases is and must be a team endeavor, involving different people serving in a wide assortment of complementary roles.

JMIR Publications and the JNT editors also recognize the value of public/private partnerships and hope the journal can support collaboration and foster sustainable innovation between these critical players. JNT aims to become a primary venue for the output of neurotechnology-focused joint ventures and more generally serve as a trusted resource for objectively reviewed and validated neurotechnology.

We explicitly want to connect these various domains, which typically are separate, and hope to do so in part by including a short, author-written statement, which we call a “handshake box” with every article. Authors of clinically oriented articles will be asked to write a handshake text that concisely articulates the technical implications of their findings, and authors of technical work will similarly write a handshake text to articulate the clinical implications of their work.

The goal of including these short, plain-language texts is to build a strong and trusted communication bridge to be shared by clinicians, caregivers, technologists, and engineers, a place to openly share, collaborate, and improve ideas and approaches. We hope to make authors feel comfortable in articulating their findings in digestible terms for all those involved in understanding neurological diseases. The worlds of all those involved in these challenges are not different and should not be separated; JNT’s handshake box is meant to close this common communication gap across domains.

## The Shapes of JNT

JNT offers 3 categories of article types.

First, JNT will welcome manuscripts in standard categories that are available in most research journals, including the JMIR family of journals, such as original papers, reviews, early reports, and commentaries. These article types will have typical formats, making it easier for authors to transfer papers between JMIR journals in cases where topics do not fall appropriately into the scope of JNT and instead are more appropriate for a different JMIR journal.

Second, you will be able to submit articles that explicitly contribute to open and reproducible science via the article types JNT Data and JNT Code. Research published as a JNT Data article should be focused on sharing data in accordance with the FAIR (Findability, Accessibility, Interoperability, and Reuse of digital assets) Principles [6]. Articles published under the JNT Code category will need to share code in a detailed and well-documented style, for example, as outlined by Jupyter Notebook [7]. These two article types will allow readers to learn from specific approaches by having details provided and explained in depth.

Third, authors will be encouraged to submit articles under the JNT Handshake article type. Besides the mandatory handshake boxes in other original work, we will welcome articles written with the specific purpose to solidify the aforementioned bridge. This article type will allow us to publish more free-form materials—perspectives, educational articles, and other narratives written by clinicians, caregivers, patients, and engineers—provided they clearly share experiences and expertise to facilitate understanding across domains.

## Welcome Aboard!

As broad and welcoming as we want JNT to be, we do have limits to our scope. JNT is not interested in fundamental neuroscience or in animal research. We aim to keep things on the applied side, where patient benefit is clear for all involved.

We warmly welcome you onto our JNT bridge and hope to see you there as part of our evolving community!

## Abbreviations

FAIR: Findability, Accessibility, Interoperability, and Reuse of digital assets
JNT: *JMIR Neurotechnology*
